# Doppler sonographic evaluation of peripheral arterial disease and its associated factors among diabetes mellitus patients at Muhimbili National Hospital, Tanzania: A hospital-based cross-sectional study

**DOI:** 10.1371/journal.pone.0328852

**Published:** 2026-04-02

**Authors:** James Alex Sumawe, Lilian Salingwa

**Affiliations:** 1 Department of Radiology, University of Dar es Salaam Mbeya College of Health and Allied Sciences (UDSM-MCHAS), Mbeya, Tanzania; 2 Department of Radiology, School of Diagnostic Medicine, Muhimbili University of Health and Allied Sciences (MUHAS), Dar es Salaam, Tanzania; Asian Institute of Medicine Science and Technology: AIMST University, MALAYSIA

## Abstract

**Background:**

Peripheral arterial disease (PAD) is more common among patients with diabetes mellitus (DM) and can lead to critical limb ischemia and amputation. Data on Doppler-diagnosed PAD are scarce in Tanzania. This study aimed to determine the prevalence of PAD and associated risk factors among DM patients referred for lower limb Doppler assessment at a tertiary hospital. Patients with ≥Grade III stenosis according to Jager’s criteria were classified as having peripheral arterial disease.

**Methods:**

Analytical cross-sectional design was conducted among 80 adult DM patients suspected of PAD at Muhimbili National Hospital between April 3, 2023, and March 30, 2024. Demographic, clinical, and sonographic data were collected. PAD was defined as ≥Grade III stenosis by Jager’s criteria. Associations between risk factors and PAD were analyzed using Firth penalized logistic regression to account for small event numbers.

**Results:**

Mean participant age was 61.2 ± 11.2 years; 55% were male. 18% had PAD, and 63.7% had hypertension. The dorsalis pedis artery was the most commonly affected segment (32.5%). Only DM duration was significantly associated with PAD (AOR = 1.19; 95% CI: 1.00–1.41; p = 0.044).

**Conclusion:**

PAD is present in a notable proportion of DM patients referred for Doppler evaluation. Longer DM duration is an exploratory predictor. Findings are limited by small sample size, few PAD events, and missing HbA1c data. Larger, multicenter studies are needed to confirm risk factors and improve generalizability.

## Introduction

Peripheral arterial disease (PAD) is the partial or complete obstruction of limb arteries, leading to reduced blood flow, tissue ischemia, and, in severe cases, ulceration or amputation [1]. Globally, PAD affects over 200 million people, and in sub-Saharan Africa (SSA) prevalence ranges from 1.7% to 52.5%, with higher rates reported when using Doppler-based diagnostics compared to clinical assessment alone [1,2].

Diabetes mellitus (DM) significantly increases PAD risk due to hyperglycemia, insulin resistance, hypertension, and Older age, smoking, obesity, and family history of cardiovascular disease further elevate risk [3–6]. PAD in DM can lead to serious complications, including non-healing ulcers, gangrene, and amputation [7,8].

While the ankle-brachial index (ABI < 0.9) is widely used internationally, it may underestimate PAD in DM due to arterial calcification [9,10]. Duplex ultrasonography provides non-invasive, accurate assessment of arterial stenosis and hemodynamic significance [7,11–13]. In this study, PAD was defined as ≥Grade III stenosis by Jager’s criteria (≥50% narrowing with hemodynamic compromise), allowing objective detection of clinically meaningful disease.

Despite increasing DM prevalence in Tanzania, local data on Doppler-diagnosed PAD are lacking. This study aimed to determine the prevalence, arterial distribution, and risk factors of PAD among adults with DM referred for lower limb Doppler sonography at a tertiary hospital. The findings will guide targeted screening and interventions to reduce PAD-related complications.

## Methods and materials

### Study design and setting

A hospital-based analytical cross-sectional study was conducted at the Radiology Department of Muhimbili National Hospital (MNH), Tanzania, between April 03, 2023 and March 30, 2024. Adult DM patients (≥18 years) with clinical suspicion of PAD referred for lower limb Doppler sonography were eligible. Patients with conditions affecting image quality (e.g., filariasis, severe swelling) or who were critically ill were excluded.

### Sample size estimation

Sample size was calculated using a finite population formula:

- Population (N) = 100 DM patients referred annually for lower limb Doppler at MNH
- Expected PAD prevalence (P) = 30.7% (Ethiopia)(12)
- 95% confidence level (Z = 1.96), margin of error (d = 0.05)

The minimum required sample size was 80 participants.

Given the small population and convenience sampling, findings are exploratory and may not be generalizable to all Tanzanian DM patients.

### Data collection

Demographic and clinical data were collected using a standardized questionnaire. Clinical variables included hypertension (measured), smoking history (lifetime, pack-years not assessed), duration of DM (from medical records), type of DM, and comorbidities. HbA1c was recorded when available but excluded from multivariable analysis due to missing values (54%).

### Doppler ultrasound

Lower limb arterial Doppler examinations were performed by trained sonographers and verified by an experienced radiologist. Ultrasound systems used included GE Healthcare and Siemens ACUSON NX3 Elite platforms, linear transducer 7–11 MHz. Arterial stenosis was graded according to Jager’s criteria:

- Grade I: 1–19% stenosis
- Grade II: 20–49% stenosis
- Grade III: 50–99% stenosis (**PAD**)
- Grade IV: 100% stenosis

### Statistical analysis

Data (S1 File) were analyzed using SPSS version 23. Descriptive statistics were used to summarize demographic, clinical, and sonographic characteristics. Binary logistic regression was first performed to estimate crude associations between independent variables and PAD. Variables with p ≤ 0.20 in crude analysis were considered for multivariable modelling. Given the small number of PAD events (n = 14), Firth’s penalized logistic regression was applied to reduce small-sample bias and mitigate separation issues. Statistical significance was set at p ≤ 0.05.

Multicollinearity among candidate predictors was assessed using variance inflation factors (VIF). All VIF values were below 2.0, indicating no evidence of problematic multicollinearity. Due to the use of penalized regression and the limited number of outcome events, conventional goodness-of-fit tests such as Hosmer–Lemeshow were not considered reliable. Therefore, model fit was primarily evaluated using penalized likelihood estimation and inspection of confidence interval width. Results should be interpreted cautiously given the small event-per-variable ratio.

### Consent

All research participants provided informed consent, a written one by signing the consent form after reading and understanding the aim of the study. A study included only adults participants (18years and above). A study did not include any minors.

### Ethical approval

The original data collection was conducted according to the guidelines of the Declaration of Helsinki and approved by Institutional Review Board of Muhimbili University of Health and Allied Sciences ethical approval numbers MUHAS-REC-03-2023-1606.

We recruited human participants for the study by distributing questionnaires which had demographic and clinic-pathological characteristics. Also data on Doppler sonographic findings was collected after performing lower limbs Doppler arterial sonography.

Start Date: 03/04/2023

End Date: 02/04/2024.

## Results

### Study participants baseline characteristics

Eighty participants were enrolled. Mean age was 61.2 ± 11.2 years; 55% were male. Most had at least primary education (53.8%) and were married (71.3%) (Table 1).

**Table 1:** Socio-demographic characteristics of the study participants, N = 80.

		*Frequency(n)/Mean + /-SD*	*Percentage(%)*
*Mean age (years)*	61.2 + /-11.2		
*Age groups (Years)*	40 and below	5	6.3
	41-60	35	43.8
	61-80	39	48.8
	Above 80	1	1.3
*Sex*	Males	44	55.0
	Females	36	45.0
*Education level*	No formal education	1	13
	Primary education	43	53.8
	Secondary education	22	27.5
	University/ college	14	17.5
*Marital status*	Single	3	3.8
	Divorced/separated	12	15.0
	Married	57	71.3
	Widow/widower	8	10.0
*Employment status*	Unemployed	22	27.5
	Employed	19	23.8
	Self employed	39	43.8

### Clinico-pathological characteristics of the study participants

Claudication and loss of sensation were the most common presenting symptoms, reported in about two-thirds of the study subjects (65% and 62.5% respectively). The majority of the study subjects (63.7%) had co-existing hypertension as a comorbid condition while approximately one-fifth admitted to having ever smoked cigarettes nearly half of the participants (46.3%) had been diagnosed with diabetes for more than 10 years. More participants had type 2 DM(95%) and the most recent value of glycated hemoglobin was abnormal in 95%.The mean value of glycated hemoglobin was 10.2 ± 5.2. More information in Table 2.

**Table 2:** Clinico-pathological characteristics of the study participants (N = 80).

*Clinical presentation*		*Frequency (n)*	*Percent (%)*
Claudication		52	65
Loss of sensation		50	62.5
Leg ulcer		50	62.5
Frank gangrene		26	32.5
Leg swelling		14	17.5
*Comorbidities/Risk factors*			
Hypertension		51	63.7
Ischaemic heart disease		9	11.2
Smoking		14	17.5
Duration of Diabetes Mellitus (Years)	10 and below	43	53.7
	Above 10	37	46.3
Type of Diabetes Mellitus	Type 1	4	5.0
	Type 2	76	95.0
*Laboratory* Most recent value of (HbA1C) in mmol/l	Below 7	19	24
	7 and above	18	22
	Not done	43	54

HbA1C- Glycated hemoglobin.

### PAD prevalence and arterial involvement

**Fig 1:**
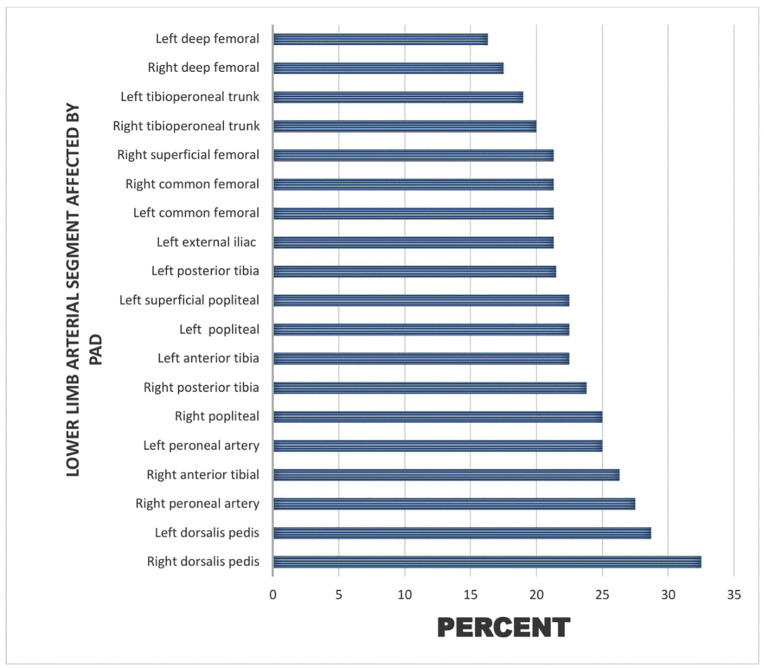
Bar chart showing lower limb arterial segment affected by PAD.

**Fig 2:**
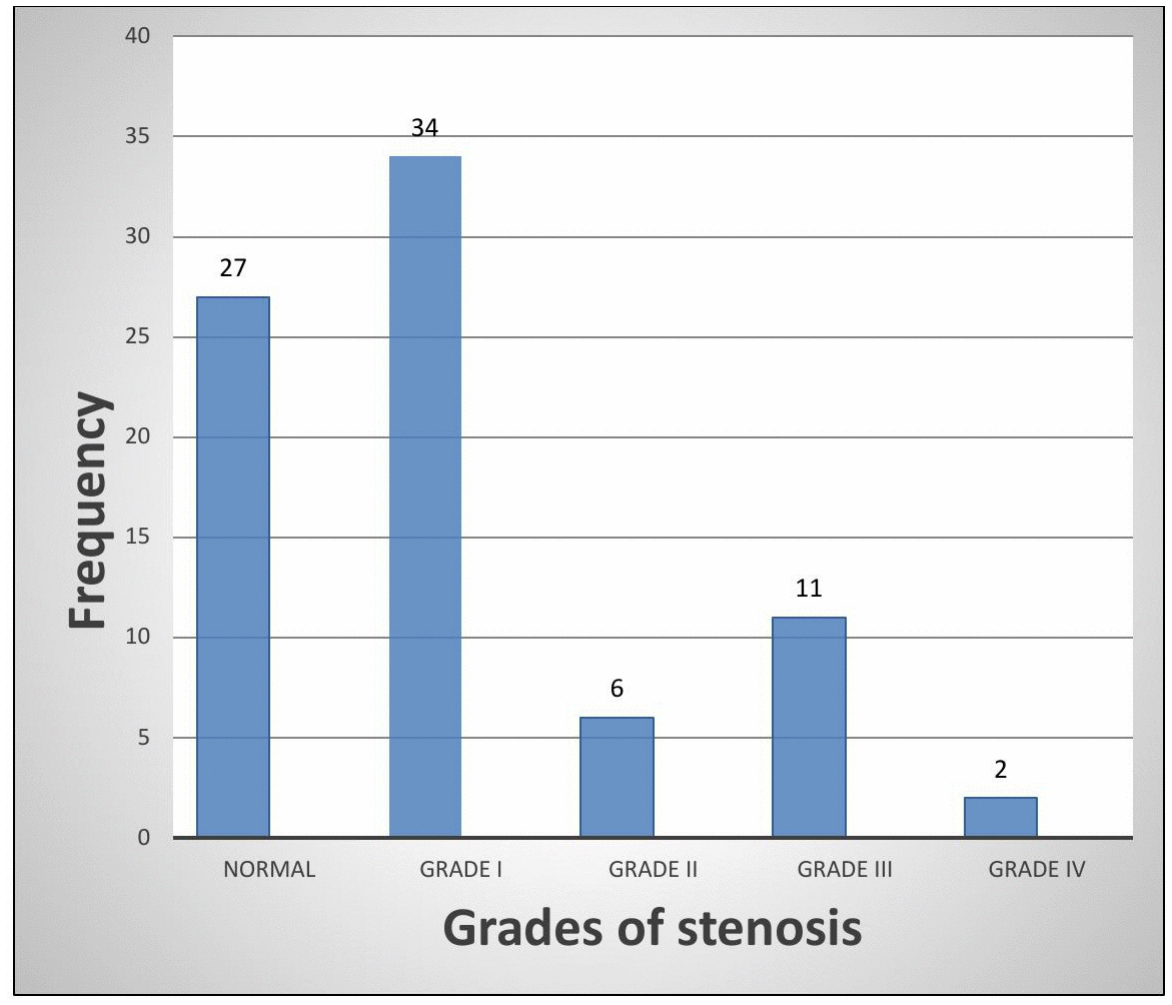
Bar chart showing grades of stenosis in the most severely affected arterial segment of the lower limb.

**Fig 3:**
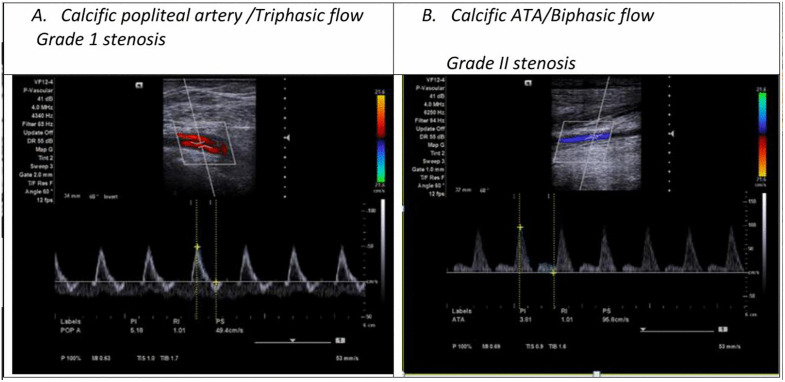
Duplex sonographic selected images extract from the two study participants.

PAD (≥Grade III stenosis) was detected in 18% (n = 14). Dorsalis pedis artery was most commonly affected (32.5%), left deep femoral artery least affected (16.3%) (Fig 1). Right and left limb involvement were equal (35% each); bilateral involvement occurred in 31%. Grade I stenosis was most frequent (42.5%); Grade IV stenosis was rare (2.5%) (Figs 2 and 3).

### Firth penalized logistic regression

In crude analysis, leg ulcer, smoking, and DM duration ≥10 years were associated with PAD. After adjustment using Firth penalized logistic regression, only DM duration remained statistically significant (AOR = 1.19, 95% CI: 1.00–1.41, p = 0.044). Other associations were not statistically significant. Confidence intervals were wide for several predictors, reflecting the limited number of PAD events and reduced statistical power. Although Firth’s method reduces small-sample bias, the low event-per-variable ratio may still contribute to instability in regression estimates. Therefore, effect sizes should be interpreted as exploratory. More information in Table 3.

**Table 3:** Firth penalized multivariable regression for PAD risk factors (N = 80).

Variable	AOR	95% CI	p-value
Leg ulcer	0.56	0.02–13.43	0.72
Frank gangrene	4.16	0.30–58.42	0.29
**DM duration >10 yrs**	**1.19**	**1.00–1.41**	0.044 *
Smoking	6.74	0.51–88.85	0.15

AOR: Adjusted odds ratio; CI: Confidence Interval; *Statistically significant.

## Discussion

This study evaluated Doppler sonographic patterns of PAD and associated factors among 80 DM patients referred for lower limb arterial assessment at a tertiary hospital in Tanzania. We found that 18% of participants met the operational definition of PAD (≥Grade III stenosis by Jager’s criteria). The dorsalis pedis artery was the most commonly affected segment, and right and left limbs were equally involved.

### Clinical presentation and comorbidities

The most frequent presenting symptoms were claudication (65%), loss of sensation (62.5%), and leg ulcers (62.5%), consistent with previous studies in Pakistan and Ethiopia [12,14]. Less than one-third of participants presented with frank gangrene or leg swelling, similar to the Pakistani study [14] but lower than some reports from Egypt [4]. Hypertension was the most common comorbidity (63.7%), higher than proportions reported in Ethiopia and Pakistan [12,14], potentially reflecting differences in population characteristics or healthcare access. Ischemic heart disease was present in 11.2% of participants, consistent with the lower prevalence reported in sub-Saharan Africa [15]. Smoking prevalence (17.5%) was comparable to Ethiopian data [12] but lower than in Dominican Republic and Egypt [4].

### Prevalence of PAD

The prevalence of PAD in our study (18%) falls within the lower range of reported prevalence in Sub-Saharan Africa. Recent meta-analyses done in 2025 [16] indicate PAD prevalence among diabetic patients in the region ranges widely from 12% to 28% [16], depending on diagnostic modality and population characteristics. Our use of duplex Doppler sonography aligns with contemporary recommendations, as ABI measurements can underestimate PAD in diabetic populations due to arterial calcification. By integrating these recent regional analyses, our findings suggest that while PAD remains an important complication of diabetes, estimates vary substantially depending on the diagnostic method and patient selection. This highlights the need for standardized, regionally adapted screening strategies.

### Factors associated with PAD

In crude analyses, leg ulcer, smoking, and DM duration ≥10 years were associated with PAD. After adjustment using Firth penalized logistic regression, only DM duration remained statistically significant (AOR = 1.19, 95% CI 1.00–1.41). This is consistent with previous studies from Ghana, Ethiopia, and Brazil [12,17,18]. Longer DM duration likely reflects cumulative vascular injury from chronic hyperglycemia, dyslipidemia, inflammation, and endothelial dysfunction [19].

Other classical well established cardiovascular risk factors such as hypertension and smoking were not significant in multivariable analysis. This may reflect limited statistical power due to small sample size (n = 80) and few PAD events (n ≈ 14), as well as missing data for HbA1c (54%). The small event-per-variable ratio also raises the possibility of overfitting and wide confidence intervals, limiting confidence in effect estimates. Our use of Firth penalized regression partially mitigates this risk, but findings should be interpreted cautiously.

In the setting of sub Saharan African countries, some studies suggested that there might be undetermined risk or epigenetic factors that may play a role in pathogenesis of PAD among patients with DM. For instance, one meta-analysis raises the suggestions than human immunodeficiency vasculopathy which is highly prevalent in sub Saharan Africa might have contributed to PAD among patients with DM [20]. This could serve as one of the population specific factors.

Importantly, the low number of PAD events (n = 14) may have resulted in unstable regression estimates despite the use of Firth penalization. Wide confidence intervals observed for several predictors suggest imprecision, and small changes in the dataset could potentially alter effect estimates. Therefore, these findings should be interpreted cautiously and viewed as hypothesis-generating rather than confirmatory.

### Strengths and Context

This study provides one of the first Doppler-based assessments of PAD in Tanzanian DM patients and highlights the distribution of arterial involvement. The study used standardized imaging protocols and experienced sonographers, ensuring reliable detection of stenosis. The findings contribute to understanding PAD burden and may inform targeted screening strategies.

## Limitations and critical reflection

Several limitations affect interpretation and generalizability. First, convenience sampling from a single tertiary hospital introduces potential selection bias, and participants may not represent the broader diabetic population in Tanzania. Second, the relatively small sample size and low number of PAD events reduced statistical power and increased the likelihood of imprecise estimates.

Third, although Firth penalized regression was used to minimize small-sample bias, the limited event-per-variable ratio may still result in unstable regression coefficients and wide confidence intervals. Fourth, missing HbA1c data (54%) prevented inclusion of glycemic control in multivariable modelling and may have obscured relevant associations. Fifth, residual confounding cannot be excluded, as socioeconomic status, medication use, diet, and physical activity were not assessed.

Finally, differences in PAD diagnostic criteria limit direct comparison with studies using ankle–brachial index (ABI < 0.9). Our Doppler-based definition (≥Grade III stenosis by Jager’s criteria) identifies hemodynamically significant disease but may yield prevalence estimates that are not directly comparable to ABI-based studies.

Given these limitations, the results should be interpreted as exploratory. Associations observed, particularly for DM duration, warrant confirmation in larger, multicenter studies.

## Conclusion

PAD affects a notable proportion of DM patients referred for lower limb Doppler evaluation, with longer DM duration being the only significant predictor in this study. However, due to small sample size, low number of events, and potential biases, these findings are **exploratory**. Clinicians should interpret the results cautiously and consider targeted Doppler screening for high-risk patients while acknowledging the limited generalizability.

### Recommendation

**Clinical:** Duplex ultrasonography remains a useful tool for assessing PAD in DM patients, particularly those with long-standing diabetes. Clinicians should consider targeted screening to identify patients at risk of complications.

**Research:** Larger, multicenter prospective studies are needed to confirm associations, improve generalizability, and explore additional risk factors, including glycemic control, medications, and lifestyle factors.

**Methodological:** Future studies should include strategies for handling missing data, ensure adequate sample size for multivariable modeling, and assess model assumptions, such as multicollinearity and fit.

## Supporting information

S1 File Data in SPSS template. (DOCX)

## Abbreviations

DM: diabetes mellitus
PAD: peripheral arterial disease
AOR: adjusted odds ratio
COR: crude odds ratio
CI: confidence interval
MNH: Muhimbili National Hospital
Hb: Hemoglobin
HbA1c: glycated hemoglobin
ABI: Ankle-Brachial Index
