# Supplementary material for: Doppler sonographic evaluation of peripheral arterial disease and its associated factors among diabetes mellitus patients at Muhimbili National Hospital, Tanzania: A hospital-based cross-sectional study
Source: PLoS One. 2026 Apr 2;21(4):e0328852. doi: 10.1371/journal.pone.0328852 (PMC13046282; doi:10.1371/journal.pone.0328852)
Supplement: S1 File — (DOCX) [file pone.0328852.s001.docx]

| **Variable** | ID_NO | age | sex |  | education_levemarital_status employment_st | | |
| --- | --- | --- | --- | --- | --- | --- | --- |
| **Label** | IDENTITY NO |  | sex |  | education levemarital status employment st | | |
|  | 1.00 |  | 76.00 | 2.00 | 2.00 | 3.00 | 1.00 |
|  | 2.00 |  | 72.00 | 2.00 | 4.00 | 4.00 | 1.00 |
|  | 3.00 |  | 74.00 | 1.00 | 4.00 | 4.00 | 1.00 |
|  | 4.00 |  | 69.00 | 1.00 | 4.00 | 3.00 | 3.00 |
|  | 5.00 |  | 47.00 | 1.00 | 3.00 | 3.00 | 2.00 |
|  | 6.00 |  | 40.00 | 2.00 | 3.00 | 2.00 | 3.00 |
|  | 7.00 |  | 39.00 | 1.00 | 3.00 | 1.00 | 3.00 |
|  | 8.00 |  | 55.00 | 1.00 | 2.00 | 3.00 | 3.00 |
|  | 9.00 |  | 35.00 | 1.00 | 2.00 | 1.00 | 3.00 |
|  | 10.00 |  | 76.00 | 2.00 | 2.00 | 3.00 | 1.00 |
|  | 11.00 |  | 41.00 | 2.00 | 2.00 | 2.00 | 3.00 |
|  | 12.00 |  | 53.00 | 2.00 | 2.00 | 2.00 | 1.00 |
|  | 13.00 |  | 57.00 | 1.00 | 3.00 | 3.00 | 3.00 |
|  | 14.00 |  | 56.00 | 2.00 | 2.00 | 3.00 | 1.00 |
|  | 15.00 |  | 74.00 | 1.00 | 4.00 | 3.00 | 3.00 |
|  | 16.00 |  | 56.00 | 1.00 | 4.00 | 3.00 | 2.00 |
|  | 17.00 |  | 43.00 | 2.00 | 4.00 | 4.00 | 2.00 |
|  | 18.00 |  | 39.00 | 1.00 | 2.00 | 3.00 | 3.00 |
|  | 19.00 |  | 65.00 | 1.00 | 2.00 | 3.00 | 3.00 |
|  | 20.00 |  | 61.00 | 1.00 | 2.00 | 3.00 | 3.00 |
|  | 21.00 |  | 59.00 | 2.00 | 2.00 | 3.00 | 3.00 |
|  | 22.00 |  | 62.00 | 3.00 | 4.00 | 1.00 | 1.00 |
|  | 23.00 |  | 40.00 | 1.00 | 2.00 | 3.00 | 3.00 |
|  | 24.00 |  | 67.00 | 2.00 | 2.00 | 3.00 | 3.00 |
|  | 25.00 |  | 54.00 | 1.00 | 2.00 | 3.00 | 3.00 |
|  | 26.00 |  | 64.00 | 1.00 | 2.00 | 3.00 | 2.00 |
|  | 27.00 |  | 59.00 | 2.00 | 2.00 | 3.00 | 1.00 |
|  | 28.00 |  | 68.00 | 1.00 | 4.00 | 2.00 | 2.00 |
|  | 29.00 |  | 57.00 | 2.00 | 4.00 | 3.00 | 2.00 |
|  | 30.00 |  | 61.00 | 2.00 | 2.00 | 3.00 | 1.00 |
|  | 31.00 |  | 49.00 | 1.00 | 2.00 | 3.00 | 3.00 |
|  | 32.00 |  | 57.00 | 1.00 | 2.00 | 3.00 | 3.00 |
|  | 33.00 |  | 98.00 | 2.00 | 2.00 | 4.00 | 1.00 |
|  | 34.00 |  | 67.00 | 2.00 | 3.00 | 2.00 | 3.00 |
|  | 35.00 |  | 68.00 | 1.00 | 2.00 | 3.00 | 3.00 |
|  | 36.00 |  | 57.00 | 2.00 | 3.00 | 3.00 | 1.00 |
|  | 37.00 |  | 59.00 | 2.00 | 2.00 | 2.00 | 1.00 |
|  | 38.00 |  | 60.00 | 2.00 | 3.00 | 3.00 | 3.00 |
|  | 39.00 |  | 73.00 | 1.00 | 2.00 | 3.00 | 3.00 |
|  | 40.00 |  | 67.00 | 1.00 | 3.00 | 3.00 | 3.00 |
|  | 41.00 |  | 47.00 | 2.00 | 2.00 | 3.00 | 2.00 |
|  | 42.00 |  | 65.00 | 1.00 | 3.00 | 3.00 | 1.00 |
|  | 43.00 |  | 56.00 | 1.00 | 2.00 | 3.00 | 2.00 |
|  | 44.00 |  | 54.00 | 2.00 | 2.00 | 3.00 | 2.00 |
|  | 45.00 |  | 64.00 | 1.00 | 2.00 | 3.00 | 2.00 |
|  | 46.00 |  | 58.00 | 1.00 | 3.00 | 4.00 | 1.00 |
|  | 47.00 |  | 54.00 | 2.00 | 2.00 | 2.00 | 2.00 |
|  | 48.00 |  | 65.00 | 2.00 | 3.00 | 2.00 | 3.00 |
|  | 49.00 |  | 75.00 | 1.00 | 2.00 | 3.00 | 2.00 |
|  | 50.00 |  | 62.00 | 2.00 | 3.00 | 3.00 | 2.00 |
|  | 51.00 |  | 64.00 | 2.00 | 4.00 | 3.00 | 1.00 |
|  | 52.00 |  | 64.00 | 1.00 | 2.00 | 3.00 | 3.00 |
|  | 53.00 |  | 63.00 | 2.00 | 3.00 | 4.00 | 1.00 |
|  | 54.00 |  | 49.00 | 1.00 | 3.00 | 3.00 | 3.00 |
|  | 55.00 |  | 74.00 | 1.00 | 4.00 | 3.00 | 2.00 |
|  | 56.00 |  | 44.00 | 2.00 | 3.00 | 2.00 | 2.00 |
|  | 57.00 |  | 53.00 | 2.00 | 3.00 | 3.00 | 3.00 |
|  | 58.00 |  | 65.00 | 1.00 | 2.00 | 3.00 | 3.00 |
|  | 59.00 |  | 62.00 | 2.00 | 2.00 | 4.00 | 3.00 |
|  | 60.00 |  | 76.00 | 1.00 | 2.00 | 3.00 | 3.00 |
|  | 61.00 |  | 59.00 | 1.00 | 2.00 | 3.00 | 4.00 |
|  | 62.00 |  | 58.00 |  |  |  |  |
|  |  |  | 48.00 |  |  |  |  |

74.00

71.00

59.00

58.00

57.00

64.00

64.00

68.00

45.00

48.00

66.00

64.00

47.00

73.00

80.00

70.00

51.00

| claudication claudication | loss_of_sensalteg_ulcer loss of sensatileg ulcer |  |  | frank_gangrenother_sympto hypertension frank gangren other symptomhypertension | |  | ischaemic_hea ischaemic hear |  |  |
| --- | --- | --- | --- | --- | --- | --- | --- | --- | --- |
| 1.00 | 2.00 |  | 1.00 | 1.00 |  | 1.00 | 1.00 |  |  |
| 1.00 | 2.00 |  | 1.00 | 2.00 |  | 1.00 | 2.00 |  |  |
| 1.00 | 2.00 |  | 2.00 | 2.00 |  | 1.00 | 2.00 |  |  |
| 2.00 | 1.00 |  | 1.00 | 1.00 |  | 1.00 | 2.00 |  |  |
| 1.00 | 2.00 |  | 1.00 | 1.00 |  | 1.00 | 2.00 |  |  |
| 1.00 | 1.00 |  | 1.00 | 1.00 |  | 2.00 | 2.00 |  |  |
| 1.00 | 2.00 |  | 1.00 | 1.00 |  | 2.00 | 2.00 |  |  |
| 1.00 | 1.00 |  | 1.00 | 2.00 |  | 1.00 | 2.00 |  |  |
| 2.00 | 1.00 |  | 1.00 | 1.00 |  | 2.00 | 2.00 |  |  |
| 1.00 | 2.00 |  | 1.00 | 2.00 |  | 1.00 | 1.00 |  |  |
| 1.00 | 1.00 |  | 1.00 | 1.00 |  | 2.00 | 2.00 |  |  |
| 1.00 | 1.00 |  | 1.00 | 2.00 |  | 1.00 | 2.00 |  |  |
| 1.00 | 1.00 |  | 1.00 | 1.00 |  | 2.00 | 2.00 |  |  |
| 1.00 | 1.00 |  | 1.00 | 1.00 |  | 1.00 | 2.00 |  |  |
| 1.00 | 1.00 |  | 1.00 | 1.00 |  | 2.00 | 2.00 |  |  |
| 1.00 | 1.00 |  | 1.00 | 2.00 |  | 1.00 | 1.00 |  |  |
| 1.00 | 1.00 |  | 2.00 | 2.00 |  | 2.00 | 2.00 |  |  |
| 1.00 | 2.00 |  | 2.00 | 1.00 |  | 1.00 | 2.00 |  |  |
| 1.00 | 1.00 |  | 1.00 | 2.00 |  | 1.00 | 2.00 |  |  |
| 1.00 | 1.00 |  | 1.00 | 2.00 |  | 2.00 | 2.00 |  |  |
| 1.00 | 1.00 |  | 1.00 | 2.00 |  | 1.00 | 2.00 |  |  |
| 2.00 | 2.00 |  | 1.00 | 2.00 |  | 1.00 | 2.00 |  |  |
| 2.00 | 1.00 |  | 1.00 | 1.00 |  | 2.00 | 2.00 |  |  |
| 1.00 | 1.00 |  | 1.00 | 1.00 |  | 1.00 | 1.00 |  |  |
| 2.00 | 1.00 |  | 1.00 | 1.00 |  | 1.00 | 2.00 |  |  |
| 1.00 | 2.00 |  | 1.00 | 2.00 |  | 1.00 | 2.00 |  |  |
| 1.00 | 1.00 |  | 2.00 | 2.00 |  | 1.00 | 2.00 |  |  |
| 2.00 | 2.00 |  | 2.00 | 2.00 leg swelling |  | 1.00 | 2.00 |  |  |
| 1.00 | 2.00 |  | 2.00 | 2.00 |  | 2.00 | 2.00 |  |  |
| 2.00 | 2.00 |  | 2.00 | 2.00 leg swelling |  | 2.00 | 2.00 |  |  |
| 2.00 | 2.00 |  | 1.00 | 1.00 |  | 1.00 | 2.00 |  |  |
| 1.00 | 1.00 |  | 1.00 | 2.00 |  | 1.00 | 2.00 |  |  |
| 2.00 | 2.00 |  | 2.00 | 2.00 leg swelling |  | 1.00 | 1.00 |  |  |
| 1.00 | 1.00 |  | 2.00 | 2.00 |  | 1.00 | 2.00 |  |  |
| 2.00 | 2.00 |  | 2.00 | 2.00 leg swelling |  | 1.00 | 2.00 |  |  |
| 2.00 | 2.00 |  | 1.00 | 2.00 |  | 1.00 | 2.00 |  |  |
| 1.00 | 1.00 |  | 1.00 | 2.00 |  | 2.00 | 2.00 |  |  |
| 2.00 | 1.00 |  | 2.00 | 2.00 |  | 1.00 | 2.00 |  |  |
| 2.00 | 2.00 |  | 2.00 | 2.00 leg swelling |  | 1.00 | 2.00 |  |  |
| 2.00 | 2.00 |  | 2.00 | 2.00 leg swelling |  | 2.00 | 2.00 |  |  |
| 2.00 | 2.00 |  | 2.00 | 2.00 leg swellling |  | 2.00 | 2.00 |  |  |
| 2.00 | 2.00 |  | 1.00 | 2.00 |  | 1.00 | 2.00 |  |  |
| 2.00 | 2.00 |  | 1.00 | 2.00 |  | 1.00 | 2.00 |  |  |
| 2.00 | 2.00 |  | 2.00 | 2.00 leg swelling |  | 1.00 | 2.00 |  |  |
| 1.00 | 1.00 |  | 2.00 | 2.00 |  | 2.00 | 2.00 |  |  |
| 2.00 | 1.00 |  | 2.00 | 2.00 |  | 2.00 | 2.00 |  |  |
| 1.00 | 1.00 |  | 1.00 | 1.00 |  | 1.00 | 2.00 |  |  |
| 1.00 | 1.00 |  | 1.00 | 1.00 |  | 1.00 | 1.00 |  |  |
| 1.00 | 1.00 |  | 1.00 | 1.00 leg swelling |  | 1.00 | 2.00 |  |  |
| 1.00 | 2.00 |  | 2.00 | 2.00 |  | 1.00 | 2.00 |  |  |
| 1.00 | 2.00 |  | 2.00 | 2.00 |  | 1.00 | 2.00 |  |  |
| 2.00 | 1.00 |  | 1.00 | 1.00 |  | 1.00 | 2.00 |  |  |
| 1.00 | 1.00 |  | 1.00 | 1.00 |  | 1.00 | 2.00 |  |  |
| 1.00 | 1.00 |  | 2.00 | 2.00 |  | 2.00 | 2.00 |  |  |
| 1.00 | 1.00 |  | 1.00 | 2.00 |  | 1.00 | 1.00 |  |  |
| 2.00 | 1.00 |  | 1.00 | 2.00 |  | 2.00 | 2.00 |  |  |
| 2.00 | 1.00 |  | 2.00 | 2.00 |  | 1.00 | 2.00 |  |  |
| 2.00 | 1.00 |  | 1.00 | 2.00 |  | 1.00 | 2.00 |  |  |
| 1.00 | 1.00 |  | 1.00 | 2.00 |  | 1.00 | 2.00 |  |  |
| 1.00 | 2.00 |  | 1.00 | 2.00 |  | 1.00 | 2.00 |  |  |
| 1.00 | 1.00 |  | 1.00 | 2.00 |  | 2.00 | 2.00 |  |  |
| smoking smoking |  | duration_of_Dtype_of_DM duration of DMtype of DM | | recent_value_sonographic_ affected_LL value of HbAIcsonographic eAffected LL | | |  |  | right_external_i right external |
|  | 2.00 | 20.00 | 2.00 |  | 1.00 | 2.00 | 2.00 |  |  |
|  | 2.00 | 3.00 | 2.00 | 8.27 | 1.00 | 3.00 | 2.00 |  |  |
|  | 1.00 | 4.00 | 2.00 |  | 2.00 |  | 2.00 |  |  |
|  | 2.00 | 20.00 | 2.00 | 9.12 | 1.00 | 1.00 | 2.00 |  |  |
|  | 2.00 | 1.00 | 2.00 |  | 1.00 | 2.00 | 2.00 |  |  |
|  | 2.00 | 8.00 | 2.00 |  | 1.00 | 1.00 | 2.00 |  |  |
|  | 2.00 | 3.00 | 2.00 |  | 1.00 | 1.00 | 2.00 |  |  |
|  | 1.00 | 11.00 | 2.00 |  | 1.00 | 2.00 | 2.00 |  |  |
|  | 2.00 | 12.00 | 1.00 | 6.50 | 2.00 |  | 2.00 |  |  |
|  | 2.00 | 23.00 | 2.00 |  | 1.00 | 2.00 | 2.00 |  |  |
|  | 2.00 | 3.00 | 2.00 |  | 1.00 | 1.00 | 2.00 |  |  |
|  | 2.00 | 23.00 | 2.00 | 25.23 | 2.00 | 1.00 | 2.00 |  |  |
|  | 2.00 | 15.00 | 2.00 |  | 1.00 | 2.00 | 2.00 |  |  |
|  | 2.00 | 13.00 | 2.00 | 8.60 | 1.00 | 2.00 | 2.00 |  |  |
|  | 1.00 | 6.00 | 2.00 | 36.37 | 2.00 | 2.00 | 2.00 |  |  |
|  | 1.00 | 10.00 | 2.00 | 20.93 | 1.00 | 2.00 | 2.00 |  |  |
|  | 2.00 | 7.00 | 2.00 |  | 2.00 |  | 2.00 |  |  |
|  | 2.00 | 12.00 | 1.00 | 15.14 | 1.00 | 1.00 | 1.00 |  |  |
|  | 2.00 | 17.00 | 2.00 |  | 1.00 | 2.00 | 2.00 |  |  |
|  | 1.00 | 3.00 | 2.00 |  | 1.00 | 1.00 | 2.00 |  |  |
|  | 2.00 | 11.00 | 2.00 | 12.69 | 1.00 | 1.00 | 2.00 |  |  |
|  | 2.00 | 8.00 | 2.00 | 9.42 | 2.00 | 2.00 | 2.00 |  |  |
|  | 2.00 | 13.00 | 1.00 | 16.76 | 1.00 | 1.00 | 2.00 |  |  |
|  | 1.00 | 27.00 | 2.00 | 17.20 | 1.00 | 3.00 | 1.00 |  |  |
|  | 2.00 | 8.00 | 2.00 |  | 1.00 | 2.00 | 2.00 |  |  |
|  | 2.00 | 12.00 | 2.00 |  | 1.00 | 1.00 | 1.00 |  |  |
|  | 2.00 | 8.00 | 2.00 |  | 2.00 | 2.00 | 2.00 |  |  |
|  | 2.00 | 15.00 | 2.00 |  | 2.00 | 2.00 | 2.00 |  |  |
|  | 2.00 | 16.00 | 2.00 |  |  | 2.00 | 2.00 |  |  |
|  | 2.00 | 2.00 | 1.00 |  | 2.00 | 2.00 | 2.00 |  |  |
|  | 2.00 | 1.00 | 2.00 |  | 1.00 | 1.00 | 2.00 |  |  |
|  | 2.00 | 13.00 | 2.00 |  | 2.00 | 2.00 | 2.00 |  |  |
|  | 2.00 | 22.00 | 2.00 |  | 1.00 | 1.00 | 2.00 |  |  |
|  | 2.00 | 17.00 | 2.00 |  | 2.00 |  | 2.00 |  |  |
|  | 2.00 | 16.00 | 2.00 |  | 1.00 | 3.00 | 2.00 |  |  |
|  | 1.00 | 10.00 | 2.00 |  | 1.00 | 1.00 | 2.00 |  |  |
|  | 2.00 | 2.00 | 2.00 |  | 2.00 |  | 2.00 |  |  |
|  | 2.00 | 3.00 | 2.00 |  | 2.00 |  | 2.00 |  |  |
|  | 1.00 | 11.00 | 2.00 |  | 2.00 |  | 2.00 |  |  |
|  | 2.00 | 10.00 | 2.00 |  | 1.00 | 2.00 | 2.00 |  |  |
|  | 2.00 |  | 2.00 |  | 2.00 |  | 2.00 |  |  |
|  | 2.00 | 15.00 | 2.00 |  | 2.00 | 2.00 | 2.00 |  |  |
|  | 2.00 |  | 2.00 |  | 1.00 | 3.00 | 1.00 |  |  |
|  | 2.00 | 8.00 | 2.00 |  | 1.00 | 2.00 | 2.00 |  |  |
|  | 2.00 | 5.00 | 2.00 |  | 1.00 | 3.00 | 1.00 |  |  |
|  | 2.00 | 10.00 | 2.00 |  | 1.00 | 3.00 | 1.00 |  |  |
|  | 2.00 | 8.00 | 2.00 |  | 1.00 | 2.00 | 2.00 |  |  |
|  | 1.00 | 5.00 | 2.00 |  | 1.00 | 2.00 | 2.00 |  |  |
|  | 2.00 | 30.00 | 2.00 |  | 1.00 | 2.00 | 2.00 |  |  |
|  | 2.00 | 12.00 | 2.00 |  | 1.00 | 3.00 | 1.00 |  |  |
|  | 2.00 | 4.00 | 2.00 |  | 1.00 | 2.00 | 2.00 |  |  |
|  | 2.00 | 3.00 | 2.00 |  | 2.00 |  | 2.00 |  |  |
| 2.00 | 17.00 |  | 2.00 | 4.48 | 1.00 | 3.00 | 1.00 |  |  |
| 2.00 | 18.00 |  | 2.00 |  | 2.00 |  | 2.00 |  |  |
| 2.00 | 25.00 |  | 2.00 | 8.30 | 1.00 | 1.00 | 2.00 |  |  |
| 2.00 | 14.00 |  | 2.00 |  | 1.00 | 2.00 | 2.00 |  |  |
| 2.00 | 12.00 |  | 2.00 | 5.57 | 1.00 | 3.00 | 2.00 |  |  |
| 2.00 | 23.00 |  | 2.00 | 11.38 | 1.00 | 2.00 | 2.00 |  |  |
| 2.00 | 17.00 |  | 2.00 |  | 1.00 | 1.00 | 2.00 |  |  |
| 1.00 | 20.00 |  | 2.00 |  | 1.00 | 3.00 | 2.00 |  |  |
| 2.00 | 10.00 |  | 2.00 | 10.32 | 2.00 |  | 2.00 |  |  |

left_extenal_illright_common_left_common_fright_superfici left_superficia right_deep_fe left_deep_femo left external ill right common fleft common feright superficialeft superficial right deep femloeft deep femor

| 2.00 | 2.00 | 1.00 | 2.00 | 1.00 | 2.00 | 1.00 |
| --- | --- | --- | --- | --- | --- | --- |
| 2.00 | 2.00 | 2.00 | 1.00 | 1.00 | 1.00 | 2.00 |
| 2.00 | 2.00 | 2.00 | 2.00 | 2.00 | 2.00 | 2.00 |
| 2.00 | 2.00 | 2.00 | 2.00 | 2.00 | 2.00 | 2.00 |
| 2.00 | 2.00 | 1.00 | 2.00 | 1.00 | 2.00 | 2.00 |
| 2.00 | 2.00 | 2.00 | 2.00 | 2.00 | 2.00 | 2.00 |
| 2.00 | 1.00 | 2.00 | 1.00 | 2.00 | 1.00 | 2.00 |
| 2.00 | 2.00 | 2.00 | 2.00 | 2.00 | 2.00 | 2.00 |
| 2.00 | 2.00 | 2.00 | 2.00 | 2.00 | 2.00 | 2.00 |
| 2.00 | 2.00 | 1.00 | 2.00 | 1.00 | 2.00 | 1.00 |
| 2.00 | 2.00 | 2.00 | 2.00 | 2.00 | 2.00 | 2.00 |
| 2.00 | 2.00 | 2.00 | 2.00 | 2.00 | 2.00 | 2.00 |
| 2.00 | 2.00 | 2.00 | 2.00 | 2.00 | 2.00 | 2.00 |
| 2.00 | 2.00 | 2.00 | 2.00 | 2.00 | 2.00 | 2.00 |
| 2.00 | 2.00 | 2.00 | 2.00 | 2.00 | 2.00 | 2.00 |
| 2.00 | 2.00 | 2.00 | 2.00 | 2.00 | 2.00 | 2.00 |
| 2.00 | 2.00 | 2.00 | 2.00 | 2.00 | 2.00 | 2.00 |
| 2.00 | 1.00 | 2.00 | 1.00 | 2.00 | 1.00 | 2.00 |
| 2.00 | 2.00 | 2.00 | 2.00 | 2.00 | 2.00 | 2.00 |
| 2.00 | 2.00 | 2.00 | 2.00 | 2.00 | 2.00 | 2.00 |
| 2.00 | 2.00 | 2.00 | 2.00 | 2.00 | 2.00 | 2.00 |
| 2.00 | 2.00 | 2.00 | 2.00 | 2.00 | 2.00 | 2.00 |
| 2.00 | 2.00 | 2.00 | 2.00 | 2.00 | 2.00 | 2.00 |
| 1.00 | 1.00 | 1.00 | 1.00 | 1.00 | 2.00 | 1.00 |
| 2.00 | 2.00 | 2.00 | 2.00 | 2.00 | 2.00 | 2.00 |
| 2.00 | 1.00 | 1.00 | 1.00 | 1.00 | 1.00 | 2.00 |
| 2.00 | 2.00 | 2.00 | 2.00 | 2.00 | 2.00 | 2.00 |
| 2.00 | 2.00 | 2.00 | 2.00 | 2.00 | 2.00 | 2.00 |
| 2.00 | 2.00 | 2.00 | 2.00 | 2.00 | 2.00 | 2.00 |
| 2.00 | 2.00 | 2.00 | 2.00 | 2.00 | 2.00 | 2.00 |
| 2.00 | 2.00 | 2.00 | 1.00 | 2.00 | 2.00 | 2.00 |
| 2.00 | 2.00 | 2.00 | 2.00 | 2.00 | 2.00 | 2.00 |
| 2.00 | 1.00 | 2.00 | 1.00 | 2.00 | 1.00 | 2.00 |
| 2.00 | 2.00 | 2.00 | 2.00 | 2.00 | 2.00 | 2.00 |
| 2.00 | 1.00 | 1.00 | 1.00 | 1.00 | 1.00 | 1.00 |
| 2.00 | 1.00 | 2.00 | 1.00 | 2.00 | 1.00 | 2.00 |
| 2.00 | 2.00 | 2.00 | 2.00 | 2.00 | 2.00 | 2.00 |
| 2.00 | 2.00 | 2.00 | 2.00 | 2.00 | 2.00 | 2.00 |
| 2.00 | 2.00 | 2.00 | 2.00 | 2.00 | 2.00 | 2.00 |
| 1.00 | 2.00 | 1.00 | 2.00 | 1.00 | 2.00 | 1.00 |
| 2.00 | 2.00 | 2.00 | 2.00 | 2.00 | 2.00 | 2.00 |
| 2.00 | 2.00 | 2.00 | 2.00 | 2.00 | 2.00 | 2.00 |
| 1.00 | 1.00 | 1.00 | 1.00 | 1.00 | 2.00 | 2.00 |
| 1.00 | 2.00 | 1.00 | 2.00 | 1.00 | 2.00 | 1.00 |
| 1.00 | 1.00 | 1.00 | 1.00 | 1.00 | 1.00 | 1.00 |
| 1.00 | 1.00 | 1.00 | 1.00 | 1.00 | 1.00 | 2.00 |
| 1.00 | 2.00 | 1.00 | 2.00 | 1.00 | 2.00 | 1.00 |
| 1.00 | 2.00 | 1.00 | 2.00 | 1.00 | 2.00 | 1.00 |
| 1.00 | 2.00 | 1.00 | 2.00 | 1.00 | 2.00 | 1.00 |
| 1.00 | 1.00 | 1.00 | 1.00 | 1.00 | 1.00 | 1.00 |
| 2.00 | 2.00 | 2.00 | 2.00 | 2.00 | 2.00 | 2.00 |
| 2.00 | 2.00 | 2.00 | 2.00 | 2.00 | 2.00 | 2.00 |
| 1.00 | 2.00 | 2.00 | 2.00 | 2.00 | 2.00 | 2.00 |
| 2.00 | 2.00 | 2.00 | 2.00 | 2.00 | 2.00 | 2.00 |
| 2.00 | 1.00 | 2.00 | 1.00 | 2.00 | 1.00 | 2.00 |
| 2.00 | 2.00 | 2.00 | 2.00 | 2.00 | 2.00 | 2.00 |
| 2.00 | 2.00 | 2.00 | 2.00 | 2.00 | 2.00 | 2.00 |
| 2.00 | 2.00 | 2.00 | 2.00 | 2.00 | 2.00 | 2.00 |
| 2.00 | 2.00 | 2.00 | 2.00 | 2.00 | 2.00 | 2.00 |
| 2.00 | 2.00 | 2.00 | 2.00 | 2.00 | 2.00 | 2.00 |
| 2.00 | 2.00 | 2.00 | 2.00 | 2.00 | 2.00 | 2.00 |
| right_popliteal left_popliteal | right_anterior_left_anterior_tiright_tibioperoleft_tibioperonright_posterior | | | | |  |
| right popliteal | left popliteal a right anterior t left anterior tibright tibiopero left tibioperoneright posterior | | | | | |
| 2.00 | 1.00 | 2.00 | 1.00 | 2.00 | 1.00 | 2.00 |
| 1.00 | 1.00 | 1.00 | 1.00 | 2.00 | 2.00 | 1.00 |
| 2.00 | 2.00 | 2.00 | 2.00 | 2.00 | 2.00 | 2.00 |
| 2.00 | 2.00 | 1.00 | 2.00 | 1.00 | 2.00 | 1.00 |
| 2.00 | 1.00 | 2.00 | 2.00 | 2.00 | 2.00 | 2.00 |
| 2.00 | 2.00 | 1.00 | 2.00 | 2.00 | 2.00 | 1.00 |
| 1.00 | 2.00 | 1.00 | 2.00 | 2.00 | 2.00 | 1.00 |
| 2.00 | 2.00 | 2.00 | 1.00 | 2.00 | 2.00 | 2.00 |
| 2.00 |  | 2.00 | 2.00 | 2.00 | 2.00 | 2.00 |
| 2.00 | 1.00 | 2.00 | 1.00 | 2.00 | 1.00 | 2.00 |
| 2.00 | 2.00 | 1.00 | 2.00 | 2.00 | 2.00 | 1.00 |
| 1.00 | 2.00 | 2.00 | 2.00 | 2.00 | 2.00 | 2.00 |
| 2.00 | 2.00 | 2.00 | 2.00 | 2.00 | 2.00 | 2.00 |
| 2.00 | 1.00 | 2.00 | 1.00 | 2.00 | 1.00 | 2.00 |
| 1.00 | 2.00 | 1.00 | 2.00 | 1.00 | 2.00 | 1.00 |
| 2.00 | 2.00 | 2.00 | 2.00 | 2.00 | 2.00 | 2.00 |
| 2.00 | 2.00 | 2.00 | 2.00 | 2.00 | 2.00 | 2.00 |
| 1.00 | 2.00 | 1.00 | 2.00 | 1.00 | 2.00 | 1.00 |
| 2.00 | 2.00 | 2.00 | 1.00 | 2.00 | 2.00 | 2.00 |
| 2.00 | 2.00 | 1.00 | 2.00 | 1.00 | 2.00 | 1.00 |
| 2.00 | 2.00 | 2.00 | 2.00 | 2.00 | 2.00 | 2.00 |
| 2.00 | 2.00 | 2.00 | 2.00 | 2.00 | 2.00 | 2.00 |
| 1.00 | 2.00 | 1.00 | 2.00 | 1.00 | 2.00 | 1.00 |
| 2.00 | 1.00 | 2.00 | 1.00 | 2.00 | 1.00 | 2.00 |
| 2.00 | 1.00 | 2.00 | 1.00 | 2.00 | 1.00 | 2.00 |
| 1.00 | 2.00 | 1.00 | 2.00 | 1.00 | 2.00 | 1.00 |
| 2.00 | 2.00 | 2.00 | 2.00 | 2.00 | 2.00 | 2.00 |
| 2.00 | 2.00 | 2.00 | 2.00 | 2.00 | 2.00 | 2.00 |
| 2.00 | 2.00 | 2.00 | 2.00 | 2.00 | 2.00 | 2.00 |
| 2.00 | 2.00 | 2.00 | 2.00 | 2.00 | 2.00 | 2.00 |
| 1.00 | 2.00 | 2.00 | 2.00 | 2.00 | 2.00 | 2.00 |
| 2.00 | 2.00 | 2.00 | 2.00 | 2.00 | 2.00 | 2.00 |
| 1.00 | 2.00 | 1.00 | 2.00 | 1.00 | 2.00 | 2.00 |
| 2.00 | 2.00 | 2.00 | 2.00 | 2.00 | 2.00 | 2.00 |
| 1.00 | 1.00 | 2.00 | 2.00 | 2.00 | 2.00 | 2.00 |
| 1.00 | 2.00 | 1.00 | 2.00 | 2.00 | 2.00 | 2.00 |
| 2.00 | 2.00 | 2.00 |  | 2.00 | 2.00 | 2.00 |
| 2.00 | 2.00 | 2.00 | 2.00 | 2.00 | 2.00 | 2.00 |
| 2.00 | 2.00 | 2.00 | 2.00 | 2.00 | 2.00 | 2.00 |
| 2.00 | 1.00 | 2.00 | 1.00 | 2.00 | 1.00 | 2.00 |
| 2.00 | 2.00 | 2.00 | 2.00 | 2.00 | 2.00 | 2.00 |
| 2.00 | 2.00 | 2.00 | 2.00 | 2.00 | 2.00 | 2.00 |
| 2.00 | 2.00 | 2.00 | 2.00 | 2.00 | 2.00 | 2.00 |
| 2.00 | 1.00 | 2.00 | 2.00 | 2.00 | 2.00 | 2.00 |
| 2.00 | 2.00 | 2.00 | 2.00 | 2.00 | 2.00 | 2.00 |
| 2.00 | 2.00 | 2.00 | 2.00 | 2.00 | 2.00 | 2.00 |
| 2.00 | 1.00 | 2.00 | 1.00 | 2.00 | 1.00 | 2.00 |
| 2.00 | 1.00 | 2.00 | 1.00 | 2.00 | 1.00 | 2.00 |
| 2.00 | 1.00 | 2.00 | 1.00 | 2.00 | 2.00 | 2.00 |
| 1.00 | 2.00 | 2.00 | 2.00 | 2.00 | 2.00 | 2.00 |
| 2.00 | 2.00 | 2.00 | 1.00 | 2.00 | 1.00 | 2.00 |
| 2.00 | 2.00 | 1.00 | 2.00 | 1.00 | 2.00 | 1.00 |
| 2.00 | 2.00 | 2.00 | 2.00 | 1.00 | 1.00 | 2.00 |
| 2.00 | 2.00 | 2.00 | 2.00 | 2.00 | 2.00 | 2.00 |
| 1.00 | 2.00 | 1.00 | 2.00 | 1.00 | 2.00 | 1.00 |
| 1.00 | 2.00 | 1.00 | 2.00 | 1.00 | 2.00 | 1.00 |
| 1.00 | 1.00 | 1.00 | 1.00 | 1.00 | 1.00 | 1.00 |
| 2.00 | 2.00 | 2.00 | 1.00 | 2.00 | 1.00 | 2.00 |
| 2.00 | 2.00 | 1.00 | 2.00 | 1.00 | 2.00 | 1.00 |
| 1.00 | 1.00 | 1.00 | 1.00 | 1.00 | 1.00 | 1.00 |
| 2.00 | 2.00 | 2.00 | 2.00 | 2.00 | 2.00 | 2.00 |

| left_posterior_tright_peronealleft_peroneal | | | right_dorsalis | left_dorsalis_pdegree_stenosVAR00001 | |  |
| --- | --- | --- | --- | --- | --- | --- |
| left posterior t right peroneal left peroneal aright dorsalis | | | | left dorsalis pedegree of stenosis in most severely afected arteria | |  |
| 1.00 | 2.00 | 1.00 | 2.00 | 1.00 | 4.00 |  |
| 1.00 | 2.00 | 2.00 | 1.00 | 1.00 | 2.00 |  |
| 2.00 | 2.00 | 2.00 | 2.00 | 2.00 | 1.00 |  |
| 2.00 | 2.00 | 2.00 | 1.00 | 2.00 | 3.00 |  |
| 1.00 | 2.00 | 2.00 | 2.00 | 2.00 | 4.00 |  |
| 2.00 | 2.00 | 2.00 | 1.00 | 2.00 | 2.00 |  |
| 2.00 | 2.00 | 2.00 | 2.00 | 2.00 | 4.00 |  |
| 2.00 | 2.00 | 2.00 | 2.00 | 1.00 | 2.00 |  |
|  | 2.00 | 2.00 | 2.00 | 2.00 | 1.00 |  |
| 1.00 | 2.00 | 1.00 | 2.00 | 1.00 | 4.00 |  |
| 2.00 | 2.00 | 2.00 | 2.00 | 2.00 | 2.00 |  |
| 2.00 | 2.00 | 2.00 | 2.00 | 2.00 | 2.00 |  |
| 2.00 | 2.00 | 2.00 | 2.00 | 1.00 | 2.00 |  |
| 1.00 | 2.00 | 2.00 | 2.00 | 2.00 | 2.00 |  |
| 2.00 | 1.00 | 2.00 | 1.00 | 2.00 | 2.00 |  |
| 2.00 | 2.00 | 2.00 | 2.00 | 1.00 | 2.00 |  |
| 2.00 | 2.00 | 2.00 | 2.00 | 2.00 | 1.00 |  |
| 2.00 | 1.00 | 2.00 | 1.00 | 2.00 | 4.00 |  |
| 2.00 | 2.00 | 2.00 | 2.00 | 1.00 | 2.00 |  |
| 2.00 | 1.00 | 2.00 | 2.00 | 2.00 | 2.00 |  |
| 2.00 | 2.00 | 2.00 | 1.00 | 2.00 | 2.00 |  |
| 2.00 | 2.00 | 2.00 | 2.00 | 2.00 | 2.00 |  |
| 2.00 | 1.00 | 2.00 | 2.00 | 2.00 | 2.00 |  |
| 1.00 | 2.00 | 1.00 | 2.00 | 1.00 | 4.00 |  |
| 1.00 | 2.00 | 1.00 | 2.00 | 1.00 | 2.00 |  |
| 2.00 | 1.00 | 2.00 | 1.00 | 2.00 | 5.00 |  |
| 2.00 | 2.00 | 2.00 | 2.00 | 2.00 | 1.00 |  |
| 2.00 | 2.00 | 2.00 | 2.00 | 2.00 | 2.00 |  |
| 2.00 | 2.00 | 2.00 | 2.00 | 2.00 | 1.00 |  |
| 2.00 | 2.00 | 2.00 | 2.00 | 2.00 | 1.00 |  |
| 2.00 | 2.00 | 2.00 | 2.00 | 2.00 | 1.00 |  |
| 2.00 | 2.00 | 2.00 | 2.00 | 2.00 | 1.00 |  |
| 2.00 | 2.00 | 2.00 | 1.00 | 2.00 | 3.00 |  |
| 2.00 | 2.00 | 2.00 | 2.00 | 2.00 | 1.00 |  |
| 2.00 | 2.00 | 2.00 | 2.00 | 2.00 | 2.00 |  |
| 2.00 | 2.00 | 2.00 | 1.00 | 2.00 | 3.00 |  |
| 2.00 | 2.00 | 2.00 | 2.00 | 2.00 | 1.00 |  |
| 2.00 | 2.00 | 2.00 | 2.00 | 2.00 | 1.00 |  |
| 2.00 | 2.00 | 2.00 | 2.00 | 2.00 | 1.00 |  |
| 1.00 | 2.00 | 1.00 | 2.00 | 1.00 | 2.00 |  |
| 2.00 | 2.00 | 2.00 | 2.00 | 2.00 | 1.00 |  |
| 2.00 | 2.00 | 2.00 | 2.00 | 2.00 | 1.00 |  |
| 2.00 | 2.00 | 2.00 | 2.00 | 2.00 | 2.00 |  |
| 2.00 | 2.00 | 2.00 | 2.00 | 2.00 | 2.00 |  |
| 2.00 | 2.00 | 2.00 | 2.00 | 2.00 | 2.00 |  |
| 2.00 | 2.00 | 2.00 | 2.00 | 2.00 | 2.00 |  |
| 1.00 | 2.00 | 1.00 | 2.00 | 1.00 | 2.00 |  |
| 1.00 | 2.00 | 1.00 | 2.00 | 1.00 | 2.00 |  |
| 2.00 | 2.00 | 2.00 | 2.00 | 2.00 | 2.00 |  |
| 2.00 | 2.00 | 2.00 | 2.00 | 2.00 | 2.00 |  |
| 1.00 | 2.00 | 1.00 | 2.00 | 1.00 | 2.00 |  |
| 2.00 | 1.00 | 2.00 | 1.00 | 2.00 | 2.00 |  |
| 2.00 | 1.00 | 1.00 | 2.00 | 2.00 | 3.00 |  |
| 2.00 | 2.00 | 2.00 | 2.00 | 2.00 | 1.00 |  |
| 2.00 | 1.00 | 2.00 | 1.00 | 2.00 | 5.00 |  |
| 2.00 | 1.00 | 2.00 | 1.00 | 2.00 | 2.00 |  |
| 1.00 | 1.00 | 1.00 | 1.00 | 1.00 | 2.00 |  |
| 1.00 | 2.00 | 1.00 | 2.00 | 2.00 | 2.00 |  |
| 2.00 | 1.00 | 2.00 | 1.00 | 2.00 | 2.00 |  |
| 1.00 | 1.00 | 1.00 | 1.00 | 1.00 | 3.00 |  |
| 2.00 | 2.00 | 2.00 | 2.00 | 2.00 | 1.00 |  |

degree of stenosis in most severely afected arterial segment:Jagers criteria

| **Variable**  **Question** | ID_NO  IDENTITY NO | age | sex  sex | education_levemarital_status education levemarital status | | employment_st employment st |
| --- | --- | --- | --- | --- | --- | --- |
| **Subquestion** |  |  |  |  |  |  |
| **Scale** | Unknown | Unknown | Unknown | Unknown | Unknown | Unknown |
| **Missings** |  |  |  |  |  |  |
| **Values/Labels** |  |  | 1 \| male  2 \| female | 1 \| no formal  2 \| primary ed  3 \| secondary  4 \| university/c | 1 \| single  2 \| divorced/s  3 \| married 3 \| self employed  4 \| widow/widower | 1 \| unemploye  2 \| employed |

| claudication claudication | loss_of_sensalteg_ulcer loss of sensatileg ulcer | | frank_gangrenother_sympto frank gangren  other symptomhypertension | |  | hypertension | ischaemic_hea ischaemic hear |
| --- | --- | --- | --- | --- | --- | --- | --- |
| Unknown | Unknown | Unknown | Unknown | Nominal | Unknown | Unknown |  |
| 1 \| yes  2 \| no | 1 \| yes  2 \| no | 1 \| yes  2 \| no | 1 \| yes  2 \| no |  | 1 \| yes  2 \| no | 1 \| yes  2 \| no |  |
| 3 \| self employed |  |  |  |  |  |  |  |

| smoking smoking | duration_of_Dtype_of_DM duration of DMtype of DM | | recent_value_sonographic_ value of HbAIcsonographic e | |  | affected_LL  Affected LL | right_external_i right external |
| --- | --- | --- | --- | --- | --- | --- | --- |
| Unknown | Unknown | Unknown | Unknown | Unknown | Unknown | Unknown |  |
| 1 \| yes  2 \| no |  | 1 \| type 1 2 \| type 2 |  | 1 \| yes  2 \| no | 1 \| right  2 \| left  3 \| both | 1 \| yes  2 \| no |  |

| left_extenal_illright_common left external ill  right common | | _left_common_fright_superfici  fleft common feright superficialeft superficial | |  | left_superficia | right_deep_fe  right deep femloeft deep femor | left_deep_femo |
| --- | --- | --- | --- | --- | --- | --- | --- |
| Unknown | Unknown | Unknown | Unknown | Unknown | Unknown | Unknown |  |
| 1 \| yes  2 \| no | 1 \| yes  2 \| no | 1 \| yes  2 \| no | 1 \| yes  2 \| no | 1 \| yes  2 \| no | 1 \| yes  2 \| no | 1 \| yes  2 \| no |  |

| right_popliteal right popliteal | left_popliteal left popliteal a | right_anterior_left_anterior_ti right anterior t  left anterior tib | | right_tibiopero right tibiopero | left_tibioperonright_posterior left tibioperoneright posterior | |
| --- | --- | --- | --- | --- | --- | --- |
| Unknown | Unknown | Unknown | Unknown | Unknown | Unknown | Unknown |
| 1 \| yes  2 \| no | 1 \| yes  2 \| no | 1 \| yes  2 \| no | 1 \| yes  2 \| no | 1 \| yes  2 \| no | 1 \| yes  2 \| no | 1 \| yes  2 \| no |

| left_posterior_ left posterior t | tright_peronealleft_peroneal right peroneal  left peroneal a | | right_dorsalis right dorsalis | left_dorsalis_pdegree_stenosVAR00001  left dorsalis pedegree of stenosis in most severely afected arteria | | |
| --- | --- | --- | --- | --- | --- | --- |
| Unknown | Unknown | Unknown | Unknown | Unknown | Unknown | Unknown |
| 1 \| yes  2 \| no | 1 \| yes  2 \| no | 1 \| yes  2 \| no | 1 \| yes  2 \| no | 1 \| yes  2 \| no | 1 \| normal  2 \| grade I  3 \| grade II  4 \| grade III  5 \| grade IV |  |

degree of stenosis in most severely afected arterial segment:Jagers criteria

| **#** | **Content** | **Reference** |  |
| --- | --- | --- | --- |
| 1 | QUESTION | [...] |  |
|  | VARIABLES | ID_NO |  |
|  | VALUES |  |  |
| 2 | QUESTION  VARIABLES | [...] | age |
|  | VALUES |  |  |
| 3 | QUESTION  VARIABLES | [...] | sex |
|  | VALUES | 1 |  |
|  |  | 2 |  |

| 4 | QUESTION | [...] |  |
| --- | --- | --- | --- |
|  | VARIABLES |  | education_level |
|  | VALUES |  | 1 |
|  |  |  | 2 |

3

4

| 5 | QUESTION | [...] |  |
| --- | --- | --- | --- |
|  | VARIABLES |  | marital_status |
|  | VALUES |  | 1 |
|  |  |  | 2 |

3

4

| 6 | QUESTION | [...] |  |
| --- | --- | --- | --- |
|  | VARIABLES |  | employment_status |
|  | VALUES |  | 1 |
|  |  |  | 2 |
|  |  |  | 3 |

| 7 | QUESTION | [...] |  |
| --- | --- | --- | --- |
|  | VARIABLES |  | claudication |
|  | VALUES |  | 1 |
|  |  |  | 2 |

| 8 | QUESTION | [...] |  |
| --- | --- | --- | --- |
|  | VARIABLES |  | loss_of_sensation |
|  | VALUES |  | 1 |
|  |  |  | 2 |

| 9 | QUESTION | [...] |  |
| --- | --- | --- | --- |
|  | VARIABLES |  | leg_ulcer |

| VALUES | 1 |
| --- | --- |
|  | 2 |

| 10 | QUESTION | [...] |  |
| --- | --- | --- | --- |
|  | VARIABLES |  | frank_gangrene |
|  | VALUES |  | 1 |
|  |  |  | 2 |

| 11 | QUESTION | [...] |  |
| --- | --- | --- | --- |
|  | VARIABLES |  | other_symptoms |
|  | VALUES |  |  |
| 12 | QUESTION  VARIABLES | [...] | hypertension |
|  | VALUES |  | 1 |
|  |  |  | 2 |

| 13 | QUESTION | [...] |
| --- | --- | --- |
|  | VARIABLES | ischaemic_heart_disease |
|  | VALUES | 1 |
|  |  | 2 |

| 14 | QUESTION | [...] |  |
| --- | --- | --- | --- |
|  | VARIABLES |  | smoking |
|  | VALUES |  | 1 |
|  |  |  | 2 |

| 15 | QUESTION | [...] |  |
| --- | --- | --- | --- |
|  | VARIABLES | ion_of_DM_since_diagnosis |  |
|  | VALUES |  |  |
| 16 | QUESTION  VARIABLES | [...] | type_of_DM |
|  | VALUES | 1 |  |
|  |  | 2 |  |

| 17 | QUESTION | [...] |
| --- | --- | --- |
|  | VARIABLES | recent_value_of_HbAIc |
|  | VALUES |  |
| 18 | QUESTION  VARIABLES | [...]  graphic_evidence_LL_PAD |
|  | VALUES | 1 |
|  |  | 2 |

| 19 | QUESTION | [...] |  |
| --- | --- | --- | --- |
|  | VARIABLES |  | affected_LL |
|  | VALUES |  | 1 |
|  |  |  | 2 |
|  |  |  | 3 |

| 20 | QUESTION | [...] |  |
| --- | --- | --- | --- |
|  | VARIABLES |  | right_external_illiac |
|  | VALUES |  | 1 |
|  |  |  | 2 |

| 21 | QUESTION | [...] |  |
| --- | --- | --- | --- |
|  | VARIABLES |  | left_extenal_illiac |
|  | VALUES |  | 1 |
|  |  |  | 2 |

| 22 | QUESTION | [...] |  |
| --- | --- | --- | --- |
|  | VARIABLES |  | right_common_femoral |
|  | VALUES |  | 1 |
|  |  |  | 2 |

| 23 | QUESTION | [...] |  |
| --- | --- | --- | --- |
|  | VARIABLES |  | left_common_femoral |
|  | VALUES |  | 1 |
|  |  |  | 2 |

| 24 | QUESTION | [...] |
| --- | --- | --- |
|  | VARIABLES | right_superficial_femoral |
|  | VALUES | 1 |
|  |  | 2 |

| 25 | QUESTION | [...] |  |
| --- | --- | --- | --- |
|  | VARIABLES |  | left_superficial_femoral |
|  | VALUES |  | 1 |
|  |  |  | 2 |

| 26 | QUESTION | [...] |  |
| --- | --- | --- | --- |
|  | VARIABLES |  | right_deep_femoral |
|  | VALUES |  | 1 |
|  |  |  | 2 |

| 27 | QUESTION | [...] |  |
| --- | --- | --- | --- |
|  | VARIABLES |  | left_deep_femoral |
|  | VALUES |  | 1 |
|  |  |  | 2 |
| 28 | QUESTION | [...] |  |
|  | VARIABLES |  | right_popliteal |
|  | VALUES |  | 1 |
|  |  |  | 2 |

| 29 | QUESTION | [...] |  |
| --- | --- | --- | --- |
|  | VARIABLES |  | left_popliteal |
|  | VALUES |  | 1 |
|  |  |  | 2 |

| 30 | QUESTION | [...] |  |
| --- | --- | --- | --- |
|  | VARIABLES |  | right_anterior_tibia |
|  | VALUES |  | 1 |
|  |  |  | 2 |

| 31 | QUESTION | [...] |  |
| --- | --- | --- | --- |
|  | VARIABLES |  | left_anterior_tibia |
|  | VALUES |  | 1 |
|  |  |  | 2 |

| 32 | QUESTION | [...] |
| --- | --- | --- |
|  | VARIABLES | right_tibioperoneal_trunk |
|  | VALUES | 1 |
|  |  | 2 |

| 33 | QUESTION | [...] |  |
| --- | --- | --- | --- |
|  | VARIABLES |  | left_tibioperoneal_trunk |
|  | VALUES |  | 1 |
|  |  |  | 2 |

| 34 | QUESTION | [...] |  |
| --- | --- | --- | --- |
|  | VARIABLES |  | right_posterior_tibia |
|  | VALUES |  | 1 |
|  |  |  | 2 |

| 35 | QUESTION | [...] |  |
| --- | --- | --- | --- |
|  | VARIABLES |  | left_posterior_tibia |
|  | VALUES |  | 1 |
|  |  |  | 2 |

| 36 | QUESTION | [...] |  |
| --- | --- | --- | --- |
|  | VARIABLES |  | right_peroneal |
|  | VALUES |  | 1 |
|  |  |  | 2 |
| 37 | QUESTION | [...] |  |
|  | VARIABLES |  | left_peroneal |
|  | VALUES |  | 1 |
|  |  |  | 2 |

| 38 | QUESTION | [...] |  |
| --- | --- | --- | --- |
|  | VARIABLES |  | right_dorsalis_pedis |
|  | VALUES |  | 1 |
|  |  |  | 2 |

| 39 | QUESTION | [...] |  |
| --- | --- | --- | --- |
|  | VARIABLES |  | left_dorsalis_pedis |
|  | VALUES |  | 1 |
|  |  |  | 2 |

| 40 | QUESTION | [...] |  |
| --- | --- | --- | --- |
|  | VARIABLES |  | degree_stenosis |
|  | VALUES |  | 1 |
|  |  |  | 2 |

3

4

5

| 41 | QUESTION | [...] |  |
| --- | --- | --- | --- |
|  | VARIABLES |  | VAR00001 |
|  | VALUES |  |  |
| * - | A special column for quickly transforming this source into ready-to-use reports, using the N'table tabulation software | |  |

**Text**

**IDENTITY NO**

**[...]**

**sex**

male

female

**education level**

no formal education

primary education

secondary education

university/college

**marital status**

single

divorced/separated

married

widow/widower

**employment status**

unemployed

employed

self employed

**claudication**

yes

no

**loss of sensation**

yes

no

**leg ulcer**

yes

no

**frank gangrene**

yes

no

**other symptoms**

open-ended verbatim

**hypertension**

yes

no

**ischaemic heart disease**

yes

no

**smoking**

yes

no

**duration of DM**

**type of DM**

type 1

type 2

**value of HbAIc**

**sonographic evidence of LL PAD**

yes

no

**Aﬀected LL**

right

left

both

**right external illiac artery**

yes

no

**left external illiac artery**

yes

no

**right common femoral artery**

yes

no

**left common femoral artery**

yes

no

**right superﬁcial femoral artery**

yes

no

**left superﬁcial femoral artery**

yes

no

**right deep femoral artery**

yes

no

**left deep femoral artery**

yes

no

**right popliteal artery**

yes

no

**left popliteal artery**

yes

no

**right anterior tibia artery**

yes

no

**left anterior tibia artery**

yes

no

**right tibioperoneal trunk artery**

yes

no

**left tibioperoneal trunk artery**

yes

no

**right posterior tibia artery**

yes

no

**left posterior tibia artery**

yes

no

**right peroneal artery**

yes

no

**left peroneal artery**

yes

no

**right dorsalis pedis artery**

yes

no

**left dorsalis pedis artery**

yes

no

**degree of stenosis in most severely afected arterial segment:Jagers criteria**

normal

grade I

grade II

grade III

grade IV

**[...]**

A special column for quickly transforming this source into ready-to-use reports, using the N'table tabulation software.

**Type / Range***

Quantity

[1 to 62]

Quantity

[35 to 98]

Single

1 to 2

1 to 1

2 to 2

Single

1 to 4

1 to 1

2 to 2

3 to 3

4 to 4

Single

1 to 4

1 to 1

2 to 2

3 to 3

4 to 4

Single

1 to 3

1 to 1

2 to 2

3 to 3

Single

1 to 2

1 to 1

2 to 2

Single

1 to 2

1 to 1

2 to 2

Single

1 to 2

1 to 1

2 to 2

Single

1 to 2

1 to 1

2 to 2

Open

Single

1 to 2

1 to 1

2 to 2

Single

1 to 2

1 to 1

2 to 2

Single

1 to 2

1 to 1

2 to 2

Quantity

[1 to 30]

Single

1 to 2

1 to 1

2 to 2

Quantity

[4,4793 to 36,37]

Single

1 to 2

1 to 1

2 to 2

Single

1 to 3

1 to 1

2 to 2

3 to 3

Single

1 to 2

1 to 1

2 to 2

Single

1 to 2

1 to 1

2 to 2

Single

1 to 2

1 to 1

2 to 2

Single

1 to 2

1 to 1

2 to 2

Single

1 to 2

1 to 1

2 to 2

Single

1 to 2

1 to 1

2 to 2

Single

1 to 2

1 to 1

2 to 2

Single

1 to 2

1 to 1

2 to 2

Single

1 to 2

1 to 1

2 to 2

Single

1 to 2

1 to 1

2 to 2

Single

1 to 2

1 to 1

2 to 2

Single

1 to 2

1 to 1

2 to 2

Single

1 to 2

1 to 1

2 to 2

Single

1 to 2

1 to 1

2 to 2

Single

1 to 2

1 to 1

2 to 2

Single

1 to 2

1 to 1

2 to 2

Single

1 to 2

1 to 1

2 to 2

Single

1 to 2

1 to 1

2 to 2

Single

1 to 2

1 to 1

2 to 2

Single

1 to 2

1 to 1

2 to 2

Single

1 to 5

1 to 1

2 to 2

3 to 3

4 to 4

5 to 5

Quantity

[...]

**Comment**
